# Proteomics, metabolomics, and ionomics perspectives of salinity tolerance in halophytes

**DOI:** 10.3389/fpls.2015.00537

**Published:** 2015-07-29

**Authors:** Asha Kumari, Paromita Das, Asish Kumar Parida, Pradeep K. Agarwal

**Affiliations:** ^1^Division of Wasteland Research, CSIR-Central Salt and Marine Chemicals Research Institute, Council of Scientific and Industrial ResearchBhavnagar, India; ^2^Academy of Scientific and Innovative Research, CSIR-Central Salt and Marine Chemicals Research Institute, Council of Scientific and Industrial ResearchBhavnagar, India

**Keywords:** halophytes, proteomics, metabolomics, ionomic, proline, glycine betaine, pinitol, 4-hydroxy-*N*-methyl proline

## Abstract

Halophytes are plants which naturally survive in saline environment. They account for ∼1% of the total flora of the world. They include both dicots and monocots and are distributed mainly in arid, semi-arid inlands and saline wet lands along the tropical and sub-tropical coasts. Salinity tolerance in halophytes depends on a set of ecological and physiological characteristics that allow them to grow and flourish in high saline conditions. The ability of halophytes to tolerate high salt is determined by the effective coordination between various physiological processes, metabolic pathways and protein or gene networks responsible for delivering salinity tolerance. The salinity responsive proteins belong to diverse functional classes such as photosynthesis, redox homeostasis; stress/defense, carbohydrate and energy metabolism, protein metabolism, signal transduction and membrane transport. The important metabolites which are involved in salt tolerance of halophytes are proline and proline analog (4-hydroxy-*N*-methyl proline), glycine betaine, pinitol, myo-inositol, mannitol, sorbitol, *O*-methylmucoinositol, and polyamines. In halophytes, the synthesis of specific proteins and osmotically active metabolites control ion and water flux and support scavenging of oxygen radicals under salt stress condition. The present review summarizes the salt tolerance mechanisms of halophytes by elucidating the recent studies that have focused on proteomic, metabolomic, and ionomic aspects of various halophytes in response to salinity. By integrating the information from halophytes and its comparison with glycophytes could give an overview of salt tolerance mechanisms in halophytes, thus laying down the pavement for development of salt tolerant crop plants through genetic modification and effective breeding strategies.

## Introduction

Soil salinity is the major abiotic stress affecting plant productivity worldwide. Many crop species, which countless people rely for survival, are negatively affected by high salinity (Wang et al., 2008a; Golldack et al., 2014). It has been estimated that 80 million ha of cultivated lands are affected by soil salinity (Zhang et al., 2012). Therefore, extensive research into plant salt tolerance has been carried out, with the aim of improving the resistance of crop plants (Ashraf and Harris, 2004). Despite advances in increasing plant productivity and resistance to a number of pests and diseases, improving salt tolerance in crop plants remains elusive, mainly because salinity affects several aspects of plant physiology. Salinity stress has a negative impact on the plant’s growth and development (Rockström and Falkenmark, 2000), that gradually decline the crop productivity. Excessive soil salinity can cause water deficit, ion toxicity, nutrient deficiency, rapid reduction in growth rate and induce many metabolic changes leading to molecular damage, thereby affecting the overall growth of salt-sensitive plants and even some halophytes (Hasegawa et al., 2000; Zhu, 2002; Flowers, 2004; Wang et al., 2007; Sobhanian et al., 2011). Salinity induced water deficit is accompanied by reduction in photosynthetic unit, production of reactive oxygen species (ROS), accumulation of various inorganic ions, and organic metabolites (Ashraf and Harris, 2004). The survival strategy of halophytes in high salt condition includes various mechanisms such as (i) selective accumulation or exclusion of ions (Mahajan and Tuteja, 2005), (ii) control of ion uptake especially K^+^ by roots and transport into leaves, since K^+^ is required for maintaining the osmotic balance, plays a role in opening and closing of stomata and is an essential co-factor for many enzymes like the pyruvate kinase (Mahajan and Tuteja, 2005), (iii) compartmentalization of ions at the cellular and whole-plant levels (Shabala and Mackay, 2011) (iv) biosynthesis of compatible solutes and osmoprotectants (Gagneul et al., 2007; Sanchez et al., 2008; Slama et al., 2015), (v) change in photosynthetic pathway (Stepien and Johnson, 2009; Uzilday et al., 2015), (vi) activation of antioxidant enzyme and synthesis of antioxidant compounds (Ozgur et al., 2013; Wang et al., 2013), (vii) synthesis of polyamines (Takahashi and Kakehi, 2010), (viii) generation of nitric oxide (NO; Luis, 2015) (xi) induction and modulation of plant hormones (Parida and Das, 2005; Gupta and Huang, 2014).

Salt tolerance is the ability of plants to grow and complete their life cycle on a substrate that contains high concentrations of soluble salt (Wang et al., 2013). Salt tolerance is defined as relative change in percent biomass production by plants in saline soil to that in non-saline soil after growth for a defined time period (Cheeseman, 1988). For uncultivated and long lived species, percent survival is used for defining the salt tolerance (Cheeseman, 1988). The level of reduction in growth and tolerance to the stress condition varies among different plant species. On this basis, plants can be categorized as glycophytes and halophytes. Halophytes, also called as salt loving plant, are plants that have the ability to withstand the salinity stress and possess salt responsive genes and proteins to counter the adverse effects of salinity (Askari et al., 2006; Yu et al., 2011), whereas, glycophytes, also referred as salinity sensitive plants, cannot tolerate the high salinity. Depending on their tolerance and demands for sodium salts, the halophytes can be distinguished as obligate and facultative halophytes. Obligate halophytes need some salt for their growth and development whereas facultative halophytes can thrive also under strict freshwater conditions. The obligate halophytes are characterized by low morphological and taxonomical diversity with relative growth rates increasing up to 50% in seawater and facultative halophytes are found in less saline habitats along the border between saline and non-saline upland and characterized by broader physiological diversity which enable them to cope with saline and non-saline conditions (Parida and Jha, 2010). The halophytes are further divided into hydro-halophytes and xero-halophytes. Hydro-halophytes grow in aquatic conditions or on wet soil. Most mangroves and salt-marsh species along coastlines are hydro-halophytes. Xero-halophytes grow in habitats where the soil is always saline and dry out periodically causing unavailability of water for the plant. Most species in desert areas are xero-halophytes and many of them are succulent. The level of salt tolerance of halophytes also differs from species to species. Less tolerant halophytes reduces their growth in saline environment to cope with salinity while show better growth on non-salinized soil (Zhu, 2001). This reduction in growth helps the plants to save energy, reduction in ROS production and decrease in demand of amino acid for protein synthesis to make free amino acids available for osmotic adjustments (Flowers, 2004). On the contrary, some halophytes flourish in high salinity condition. They are termed as extremophiles (Kosová et al., 2013). The increasing damage caused by high salinity has encouraged widespread research on halophyte responses to salinity and their adaptive mechanisms (Zhu, 2001; Munns and Tester, 2008). In the course of evolution, halophytes have developed various physiological and biochemical mechanisms which are responsible for regulating growth and development to ensure their survival in high salt environment (Flowers and Colmer, 2015). The genetic, physiological and biochemical properties which enable halophytes to cope with high salinity conditions are at the moment subject of intense research. Although plant breeders have successfully improved salinity tolerance of some crops in recent decades, using plant vigor or seed yield as the main selection criteria, selection may be more convenient and practicable if the crop possesses distinctive indicators of salt tolerance at the whole plant, tissue or cellular level. Thus, there is a need to understand the underlying proteomic, metabolomic, and ionomic prospect of salinity tolerance so as to provide plant breeders and genetic engineers with appropriate indicators. Halophytes are viable organisms for studying the mechanisms they use to simultaneously handle high salinity because they are naturally selected in saline dry environments.

Proteomics and metabolomics are two important “–omic” techniques in the post-genomic era (Fernandez-Garcia et al., 2011). Proteomics is the study of large-scale proteins in an organism encoded by its genome (Zhang et al., 2013). Proteomics is a powerful tool for describing complete proteomes at organelle, cell, organ or tissue levels. It can also be used to compare proteomes under various conditions of stress like exposure to high salinity (Witzel et al., 2009; Fernandez-Garcia et al., 2011). Metabolomics focuses on a global profile of the low molecular weight (1000 Da) metabolites which are the end products of metabolisms in biofluids, tissues and even whole organism (Brosché et al., 2005). The characterization of endogenous metabolites can then provide the information of metabolic status in an organism to assess the biological responses induced by exogenous factors (Liu et al., 2011). Both proteomics and metabolomics are frequently used to characterize the perturbations in metabolic pathways and corresponding enzymes and stress-responsive proteins induced by exogenous factors (Zhang et al., 2012). Ionomics is the study of the ionome, involves the simultaneous and quantitative measurement of the elemental composition of organisms or tissues and changes in this composition in response to physiological processes (Salt et al., 2008). Obviously, a combination of omic techniques like proteomics, metabolomics, and ionomics could potentially validate and complement one another, while analyzing the effects of environmental stress factors like high salinity in organisms. Halophytes may have numerous efficient adaptive mechanisms to survive and complete their life cycle under stressful conditions. Thus, halophytes are plants of great importance for proteomic, metabolomic, and ionomic studies to unravel their salt tolerance mechanisms with the longer term aim of transferring the tissue tolerance trait to commercial crops which have much lower salt tolerance efficacy.

In this context many researchers have reported differential regulation of proteins, metabolites and ions related to salinity stress. Wang et al. (2014), have investigated on proteome-level changes in *Halogeton glomeratus* which when subjected to NaCl stress revealed the abundance levels of four antioxidative enzymes *viz.* Fe-superoxide dismutase (Fe-SOD), Cytosolic APX and phospholipid hydroperoxide glutathione peroxidase (PHGPX) under salt conditions to regulate the balance of ROS formation and removal to defend against oxidative stress and cell damage. Similarly, Sobhanian et al. (2010) have reported the down-regulation of photosynthesis-related proteins in *Aeluropus lagopoides*, a halophyte C_4_ plant and metabolome studies have indicated the up-regulation of amino acids and down-regulation of TCA cycle related metabolites. Similarly, Tada and Kashimura (2009) have suggested that FBP aldolase and osmotin play important roles in salt tolerance. Also, enzymes of EMP pathway *viz*, fructokinase-1 (FRK) and 2, 3-bisphosphoglycerate-independent PGAM have been reported to up-regulate under NaCl stress condition in the leaves of Mangrove *Kandelia candel* (Wang et al., 2014). Ion related salt tolerance includes increased activity and amount of plasma membrane ion transporters (SOS1) for salt ion exclusion, enhanced activity of tonoplast ion transporters NHX (Yuan et al., 2015), ion transporters H^+^ ATPase and FBP aldolase for intracellular salt ion compartmentation and blockage of Na permeable tonoplast SV and FV channels for prevention of Na^+^ back-leak into cytosol (Tada and Kashimura, 2009; Wakeel et al., 2011; Bonales-Alatorre et al., 2013). Choline and polyamines have been reported to control the activities of SV channels to prevent the back leakage of sodium (Pottosin and Shabala, 2014; Pottosin et al., 2014). Along with these, increased lignification of xylem vessels in *Salicornia europaea* has been reported for long distance transport of excluded salt ion through transpiration stream (Wang et al., 2007).

In accordance with the change in the protein profile of halophytes in response to salinity the metabolic profile also changes for better adaptation in the saline environment. Many earlier studies have indicated up-regulation and down-regulation of certain metabolites in halophytes, *viz*, Brosché et al. (2005) have reported a study on the salt-tolerant tree *Populus euphratica* which revealed increase in amino acid levels, specifically proline, valine, and β-alanine along with changes in sugar and polyol metabolism. The levels of glycerol, glyceric acid, and myo-inositol have increased while those of fructose and mannitol decreased slightly. Similarly, Gong et al. (2005) have investigated that sugars, e.g., sucrose, glucose, and fructose, along with proline, citric acid, malic acid, and succinic acids have been constitutively higher in *Thellungiella halophila* than in *Arabidopsis thaliana*. Also, the raffinose-pathway metabolites, raffinose, myo-inositol and galactinol have accumulated to a greater extent in *T. halophila* than in *A. thaliana* in response to salt stress, while malic, fumaric, aspartic and phosphoric acid levels have decreased to a greater extent in the halophyte. The proteomic, metabolomics, and ionomic studies in different halophytes in response to high salinity by many other researchers have been discussed later in this review.

### Halophytes versus Glycophytes an Overview

**FIGURE 1:**
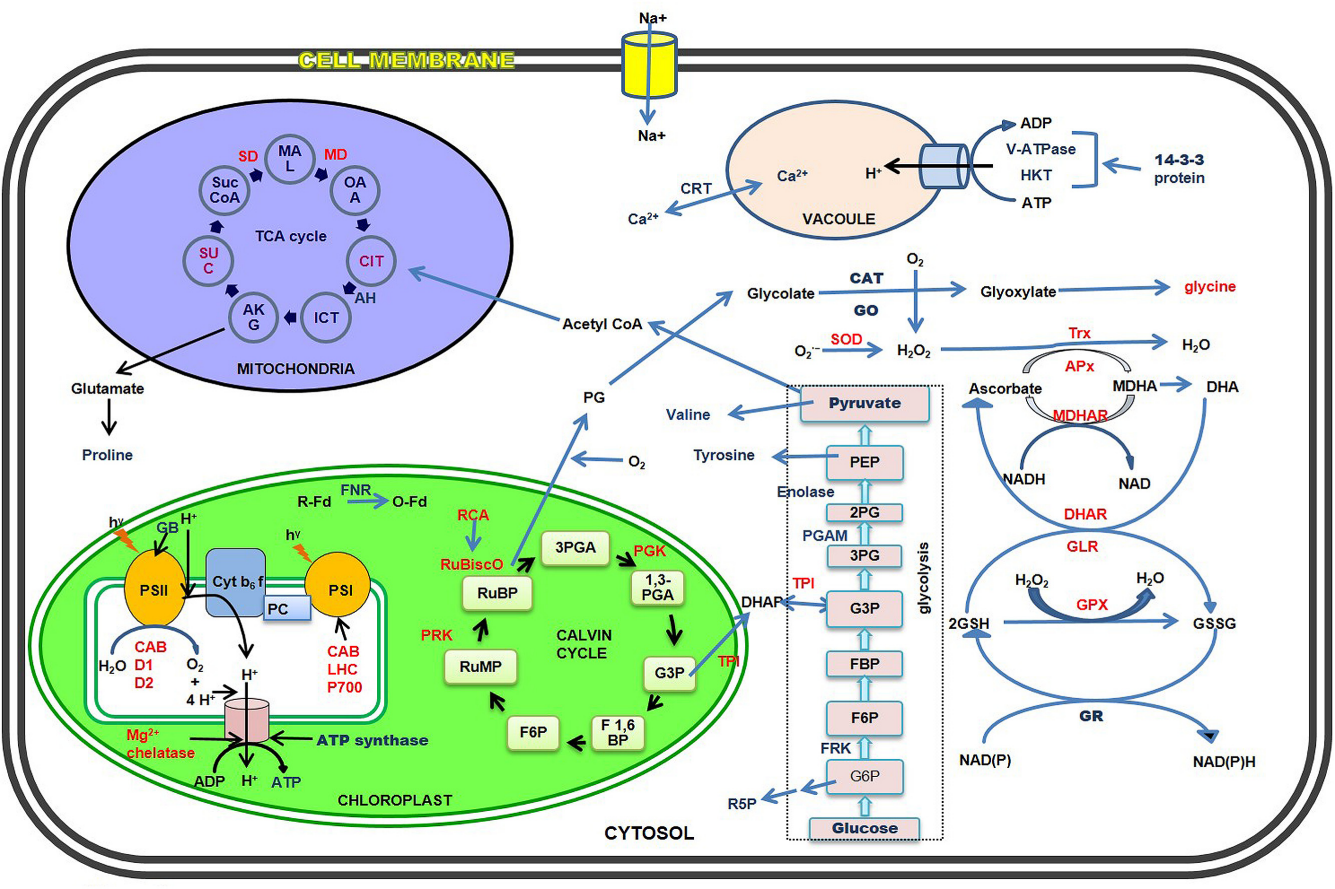
**Schematic representation of major salt stress-responsive proteins and metabolites of cytosol, chloroplasts, mitochondria, and vacuoles in the halophytes.** The up- and down-regulated proteins and metabolites have been shown in blue and red fonts respectively.

Salinity has profound effect on halophytes and glycophytes proteome, metabolome, and ionic profiles. Every plant has an optimal level for salt tolerance. The intensity of the salt stress is directly proportional to the complexity of the plant’s response to it. According to level of salt tolerance, plants can be divided into halophytes and glycophytes. Halophytes, which are exceptional, have a high salt-tolerance to even 1000 mM NaCl concentration (Flowers and Colmer, 2008). Such plant’s mechanism includes complex physiological, morphological, molecular and biochemical changes (Wang et al., 2001), which protects them under high salt/NaCl stress (**Figure 1**). Roots play a primary role for any particular changes that occurs in the plant. In normal conditions, roots absorb the water and other essential nutrients from the soil and supply them to the leaves and to the essential parts of the plant. But in salinity conditions, Na^+^ is absorbed more instead of water, as it is abundant in the soil. This creates an ionic disbalance across the membrane, hence disturbs the normal functioning of the plant which leads to either tolerancy or susceptible to death. Glycophytes which are sensitive to salt stress also adapts under low salt concentrations (>100 mM NaCl). Mechanisms very similar to those that seem to function in stress protection in the halophytes have emerged from the analysis of glycophytic species, supporting the fact that stress tolerance mechanisms are ubiquitous. Since, stress tolerance is a multigenic trait; the molecular and biochemical pathways leading to products or processes that improve tolerance are likely to act additively and synergistically.

A comparative proteomics study between halophyte (*T. halophila*) and glycophyte (*A. thaliana*) by Pang et al. (2010) has revealed that the advantages of halophytes over glycophytes may result simply from the more efficient performance of a few basic biochemical tolerance mechanisms. In *T. halophila*, an up-regulation of RCA and enzymes involved in carbon assimilation have been found while in *A. thaliana*, a down-regulation of carbon assimilation (decrease of RCA and Calvin cycle activity) have been observed under salinity stress. Similarly, accumulation of several proteins involved in protein biosynthesis, namely ribosomal proteins and proteins involved in translational apparatus (eIF3A) have been found in *T. halophila* than in *A. thaliana*. A similar comparative study on common wheat (*Triticum aestivum*) and halophytic hybrid *Thinopyrum ponticum* have revealed that eIF5A3, which plays important role in altering the cell cycle to reduce senescence, has been abundant in *Thinopyrum ponticum* as compared to wild parent, thus indicating anti- senescence ability of hybrid under high salt conditions (Wang et al., 2008a; Peng et al., 2009). Also, an increased abundance of RuBisCO subunits and vacuolar proton ATPase (V-ATPase) subunit E along with enhanced accumulation of Chlorophyll ab binding protein (CP24) protein precursor for stabilization of PSII have been found in the salt tolerant hybrid (Peng et al., 2009). In the same context, Sengupta and Majumder (2009) have carried out comparative proteomic study on rice cultivars Pokkali, IR64 and halophytic wild rice (*Porteresia coarctata*). In this study, they have found a heightened level of proteins involved in protection and stabilization of PSI and PSII under salt stress which involved oxygen evolving complex (OEC), chlorophyll binding protein CP47, Rubisco large sub unit (Rubisco LSU) and RCA. In addition, *Porteresia* have shown higher levels of myo-inositol-1-phosphate synthase and glutamine synthetase (GS). These enzymes are involved in osmolyte biosynthesis and a dehydration-responsive CRT/DRE binding protein which belongs to transcription factors inducing expression of several late embryogenesis abundant (LEA) proteins. Along with the above mentioned, an increased level of cellulose synthase have been found in *Porteresia*, which indicates an adaptation to high salt concentration as to cope with enhanced risk of adverse structural changes in cell wall structure due to replacement of Ca^+^ by Na^+^. At last, a profound increase in energy-saving enzymes such as sucrose synthase (SUS) has been reported. SUS leads to production of Uridine diphosphate (UDP) glucose in place of invertase leading to glucose, a perfect adaptation strategy by *Porteresia* for saving energy under salt stress, in order to use energy for an active stress tolerance (Sengupta and Majumder, 2009). Thus, interpretation of all the comparative studies between halophytes and glycophyte suggest that halophytes are salt tolerant due to their ability to maintain low cytoplasmic Na^+^ concentration even in an ambient salt environment (Wang et al., 2008b; Sengupta and Majumder, 2009; Pang et al., 2010) by efficient Na^+^ compartmentalization and exclusion than glycophytes.

## Proteomic Studies in Halophytes

Proteomics permit us to understand proteins and their modifications which may not be echoed by analysis of gene expression (Zhang et al., 2013). The term proteomics can be defined as the global portrayal of complete set of proteins present in a biological sample. Proteomics has presently gained more attention because it permits the quantitative and qualitative valuation of a broad-spectrum of proteins that can be related to specific cellular responses (Pérez-Clemente et al., 2013; Zhang et al., 2013; Edwards et al., 2014). Added to this, proteomics can be used not only to study expression profiling of the whole cell, but also can apply to study of cellular compartments and organelles and their time-resolved dynamics (Thelen, 2007). Thus, the objective of proteomics is to attain a comprehensive and assimilated view of biology by cramming all the proteins of a cell rather than each one individually (Angel et al., 2012). Proteomics have been extensively used for studying protein profiles of plants under stress (**Table 1**) to reveal the specific genes and proteins contributing to the salt tolerance and survival of the halophytes in saline conditions (Wang et al., 2014). Using the proteomic technologies many proteins related to salt tolerance have been identified and the related genes are cloned and transferred to glycophytes like tobacco, rice, *Arabidopsis* to improve salt tolerance (Tang et al., 2011). Salt stress-related proteomic analyses conducted in many halophytes such as *Suaeda aegyptiaca* (Askari et al., 2006), *Bruguiera gymnorrhiza* (Tada and Kashimura, 2009), *Salicornia europaea* (Wang et al., 2009; Fan et al., 2011), *Aeluropus lagopoides* (Sobhanian et al., 2010), *Cakile maritima* (Debeza et al., 2012), *Puccinellia tenuiflora* (Yu et al., 2011), *Thellungiella halophila* (Wang et al., 2013), *K. candel* (Wang et al., 2013), *Halogeton glomeratus* (Wang et al., 2014), have identified plentiful salt-responsive proteins which contribute to various functions such as photosynthesis, osmotic and ionic homeostasis, signal transduction, ROS scavenging systems etc. (Zhang et al., 2012). Role of different proteins in conferring salt tolerance are discussed in **Figure 1**, **Table 2** and below:

**Table 1:** Proteomic studies in different halophytes in response to various levels of salinity.

Plants	Salinity levels	Proteomic techniques	Reference
*Suaeda aegyptiaca*	0, 150, 300, 450, and 600 mM	2-DE LC/MS/MS	Askari et al. (2006)
*Thinopyrum ponticum*	50, 100, 150, and 200 mM	2-DE MALDI-TOF/TOF	Wang et al. (2008a)
*Salicornia europaea*	0, 200, 600, and 800 mM	2-DE MALDI-TOF/TOF	Wang et al. (2009)
*Bruguiera gymnorrhiza*	50, 100, 150, 200, and 500 mM	2-DE LC–MS/MS	Tada and Kashimura (2009)
*Aeluropus lagopoides*	150, 450, 600, and 750 mM	2-DE Edman sequencing nanoLC–MS/MS	Sobhanian et al. (2010)
*Thellungiella halophila*	0, 50, and 150 mM	2-DE MALDI-TOF/TOF nano ESI-MS/MS	Pang et al. (2010)
*Aster tripolium*	0, 50, 150, and 300 mM	2-DE MALDI-TOF/TOF	Ueda et al. (2003)
*Suaeda fruticosa*	0, 50, 100, and 200 mM	2-DE LC–MS/MS	Khan (2011)
*Hordeum marinum*	100, 200, and 300 mM	MALDI-TOF-TOF/MS	Garthwaite et al. (2005)
*Kandelia candel*	0 and 600 mM	MALDI-TOF-TOF/MS	Wang et al. (2014)
*Porteresia coarctata*	200 and 400 mM	LC–MS/MS	Sengupta and Majumder (2009)
*Puccinellia tenuiflora*	0, 50, and 150 mM	LC-MS/MS analysis	Yu et al. (2011)
*Physcomitrella patens*	250, 300, and 350 mM,	Native gel electrophoresis	Wang et al. (2008b)

**Table 2:** A list of major up- and down-regulated functional proteins involved in salt tolerance in different halophytic species.

Functional proteins	Plant species	Regulation		Function	Reference
		Up	Down		
IMT1	*Mesembryanthemum crystallinum* (Ice plant)	↑	–	Produces D-ononitol, which is further epimerized to D-pinitol	Vernon and Bohnert (1992), Bohnert et al. (1994)
Major Intrinsic Proteins (MIPs)	*M. crystallinum* (Ice plant)	–	↓	Involved in water flux	Yamada et al. (1995)
OEE	*Bruguiera gymnorrhiza*	↑	–	Repair the injury of the PSII complex	Tada and Kashimura (2009)
	*Aeluropus lagopoides*	↑	–		Sobhanian et al. (2010)
	*Puccinellia tenuiflora*	–	↓		Yu et al. (2011)
HSP70	*B. gymnorrhiza*		–	Repair the injury of the PSII complex	Tada and Kashimura (2009)
	*Tangut nitraria*	↑	–	folding of nascent chain polypeptides	Cheng et al. (2015)
	*A. lagopoides*	↑	–	import/translocation of mitochondrial or chloroplast precursor proteins	Sobhanian et al. (2010)
HSP60	*Physcomitrella patens*	↑	–	transport of proteins from the cytoplasm to mitochondrial matrix, refolding of proteins	Wang et al. (2008b)
PGAM	*Kandelia candel*	↑	–	Enzymes of EMP pathway	Wang et al. (2014)
FRK	*K. candel*	↑	–	Enzymes of EMP pathway	Wang et al. (2014)
Rubisco LSU and RCA	*Salicornia europaea*	↑	–	ROS scavengimg, calvin cycle	Wang et al. (2009), Fan et al. (2011)
	*Porteresia coarctata*	↑	–	Calvin cycle	Sengupta and Majumder (2009)
	*P. tenuiflora*	↑	–	Ros scavenging, calvin cycle	Yu et al. (2011)
	*Dunaliella salina*	↑	–	Photosynthesis related protiens	Liska et al. (2004)
CAB	*A. lagopoides*	↑	–	PSI	Sobhanian et al. (2010)
	*Cakile maritima*	↑	–	PSI	Debeza et al. (2012)
	*P. patens*	–	↓	PSI	Wang et al. (2008b)
	*T. nitraria*	–	↓	PSI	Cheng et al. (2015)
Proline dehydrogenase (PDH)	*Thellungiella halophila*	–	↓	Proline degradation	Wang et al. (2013)
D2 protein	*Suaeda aegyptiaca*	↑	–	PSII	Askari et al. (2006)
	*A. lagopoides*	↑	–	PSII	Sobhanian et al. (2010)
D1	*P. tenuiflora*	–	↓	PSII assembly/stabilization	Yu et al. (2011)
APX, Fe-SOD, PHGPX	*Halogeton glomeratus*	↑	–	Antioxidative	Wang et al. (2014)
light harvesting complex (LHC) CABs	*K. candel*	–	↓	PSI	Wang et al. (2013)
	*P. tenuiflora*	–	↓	PSI	Yu et al. (2011)
	*P. coarctata*	↑	–	chlorophyll a/b binding	Sengupta and Majumder (2009)
	*P. patens*	↑	–	chlorophyll a/b binding	Wang et al. (2008b)
	*T. nitraria*	↑	–	PSI	Cheng et al. (2015)
SOD	*S. europaea*	↑	–	conversion of O_2_ to H_2_O and O_2_	Wang et al. (2009)
	*K. candel*	↑	–	Antioxidant properties	Wang et al. (2013)
	*T. nitraria*	↑	–	Antioxidant properties	Cheng et al. (2015)
	*P. tenuiflora*	↑Upto 50 mM NaCl	↓150 mM NaCl	Antioxidant properties	Yu et al. (2011)

### Proteins Involved in Photosynthesis

In response to salinity stomatal conductance decreases as the stomatal aperture is sensitive to salinity. This closure of stomata leads to decline in intracellular CO_2_ in the leaves causing decrease in photosynthesis (Centritto et al., 2003; Redondo-Gómez et al., 2007). In *Puccinellia tenuiflora*, it has been reported that photosynthetic rate, stomatal conductance, intercellular CO_2_ and transpiration rate decreased with increasing salinity, whereas water usage efficiency increased (Yu et al., 2011). Similar findings were shown in *Tangut nitraria* (Cheng et al., 2015) and *K. candel* (Wang et al., 2013). Proteins related to photosynthesis can be grouped into proteins related to photosystem and proteins of Calvin cycle. Their regulation under salt stress has been discussed here:

#### Photosystem Related Proteins

Proteomic analysis revealed that several proteins associated with photosynthesis have differentially expressed upon salt stress. Previous studies have reported that OEE, consisting of OEE1, OEE2, and OEE3, play an important role in the light-induced oxidation of water in PSII of plants (Sugihara et al., 2000). However, these subunits in PSII complex can be easily dissociated under salt stress. Up-regulation of OEE2 has been reported in *B. gymnorrhiza* in response to salt stress, which suggests that it might be needed to repair the injury of the PSII complex and to maintain the oxygen evolution reaction (Tada and Kashimura, 2009). Similarly, Sobhanian et al. (2010) reported that OEE and PEP expression is increased in *Aeluropus lagopoides.* On the contrary, OEE1 expression was reported to be down regulated in *P. tenuiflora* (Yu et al., 2011). CAB 2, a component of the light-harvesting complex of PSI, facilitates light absorption and transfer of the excitation energy to reaction centers for the reduction of NADP to NADPH (Wan and Liu, 2008). This protein downregulation have been reported in *Puccinellia tenuiflora* (Yu et al., 2011), *Physcomitrella patens* (Wang et al., 2008b) and *Tangut nitraria* (Cheng et al., 2015). On the contrary, its accumulation has increased in *Aeluropus lagopoides* (Sobhanian et al., 2010). In a similar manner, some PSII assembly/stabilization-related proteins expression for example, D1 have declined in *Puccinellia tenuiflora* (Yu et al., 2011), but accumulation of D2 protein have increased in *Aeluropus lagopoides* (Sobhanian et al., 2010). Furthermore, two thylakoid lumen (TL) proteins, TL19 and TL18.3 have been reported to decrease in *A. thaliana* and *T. halophila* respectively (Pang et al., 2010; Zhang et al., 2012). TL18.3 regulates turnover of D1 and the assembly of PS II (Sirpio et al., 2007) whereas, TL19 is a member of PS I. It functions in the oxidation of PC (Hippler et al., 1989). The decrease in level of TL19 and TL18.3 suggest that salinity causes damage to thylakoid, thus reducing photosynthesis.

#### Calvin Cycle Proteins

Calvin cycle related protein like, Rubisco large subunit, Rubisco large subunit-binding protein subunit beta and Rubisco subunit binding-protein alpha subunit, all have significantly increased in abundance under salt stress. Rubisco is a stomatal protein responsible for CO_2_ fixation. Rubisco content in leaves is proportional to photosynthesis rate, thus its decrease shows decrease in photosynthetic rate. Rubisco subunit binding-protein alpha subunit is important in Rubisco complex assembly. Rubisco large subunit-binding protein subunit beta (RUBA) is related to Rubisco activation (Komatsu et al., 2009). A salt-induced increase in the level of Rubisco LSU, Rubisco small sub unit (SSU) as well as LHC CAB has been detected in *Physcomitrella patens* gametophyte (Wang et al., 2008b) and in *Porteresia coarctata* leaves (Sengupta and Majumder, 2009). On the contrary, Rubisco LSU, Rubisco SSU activity has decreased slightly and that of RCA activity have decreased severely in *Aeluropus lagopoides* (Sobhanian et al., 2010). Similar findings have been shown in *Puccinellia tenuiflora* (Yu et al., 2011), *K. candel* (Wang et al., 2013), and *Tangut nitraria* (Cheng et al., 2015). The chloroplast-localized protein TK, which is involved in the regeneration phase of the Calvin cycle, has also been up-regulated under salt stress in *K. candel* (Wang et al., 2013) and *P. tenuiflora* (Yu et al., 2011). In addition, the up-regulation of the Calvin cycle produces more photosynthetic products, such as sucrose and starch, to improve plant salt tolerance (Wang et al., 2013). In addition to this, PRK level have decreased in *P. tenuiflora* (Yu et al., 2011), *A. lagopoides* (Sobhanian et al., 2010) but on the other hand increased in *Porteresia coarctata* (Sengupta and Majumder, 2009) and *Salicornia europaea* (Wang et al., 2009).

### Proteins Related to Carbohydrate and Energy Metabolism

Large amount of energy is needed for the growth and development of plants under salt stress. This energy is mainly produced through carbohydrate metabolism, such as glycolysis (EMP) and TCA cycle (Höper et al., 2006). 2,3-bisphosphoglycerate-independent PGAM and fructokinase-1 (FRK) are enzymes of EMP. PGAM have been up regulated up to 600 mM NaCl stress while FRK have reached maximum abundance under 450 mM salt concentration in *K. candel* (Wang et al., 2013). FRK catalyzes the transfer of a phosphate group from ATP to fructose in glycolysis and is the most important gateway in the control of sugar influx into EMP (Höper et al., 2006). Furthermore, three more proteins representing AH, SD, and MD have found to be up-regulated. The first two enzymes are key enzymes in the TCA cycle. Thus, the increase of FRK under NaCl along with the other enzymes of EMP-TCA would contribute to glucose breakdown for energy generation to cope with salt stress. In response to high salt concentration up regulation of these proteins have been reported in *Puccinellia tenuiflora* (Yu et al., 2011) and *K. candel* (Zhao et al., 2013). In addition to these, glycolysis related protiens like Triosphosphate isomerase, glyceraldehyde-3-phosphate dehydrogenase, PGK and enolase has been up regulated in *Aeluropus lagopoides* (Sobhanian et al., 2010) and *Physcomitrella patens* gametophyte (Wang et al., 2008b). In addition, mitochondrial F1–ATPase beta subunit, ATP synthase CF1 alpha subunit, and F1 ATPase, involving in ATP synthesis, have up-regulated under salt stress (Wang et al., 2008b). Salinity modulates electron transfer efficiency and electrochemical proton gradients across the membranes, affecting ATP synthesis and NADPH formation. Thus, the energy requirements in response to salinity may considerably increase. The up-regulated ATP synthesis indicates that adjustment of ATP formation and its utilization for synthesis of compatible solutes is one of the strategies of plants to cope with salt stress (Zhang et al., 2013). Enhance in activity of ATPase have been reported in *Aeluropus lagopoides* and *Physcomitrella patens* (Wang et al., 2008b; Sobhanian et al., 2010).

The results indicated that EMP-TCA activity coupling with ATPase in halophytes have enhanced, suggesting that the respiratory metabolism in halophytes have increased under salt stress. Increased EMP-TCA activity and ATP synthesis imply that salt stress forces the plant to remobilize energy to cope with salt stress. Together with the amplification of the light reactions of photosynthesis, this may insure the continuous generation of ATP and NAD(P)H necessary to mediate the enhanced salt resistance in halophytes. Thus, they seem to derive its ability to improve stress tolerance through the adjustment of its-energy metabolism (Zhao et al., 2013).

### Proteins Associated with Detoxification and Antioxidation

Salinity effects plant in two different ways. First, it causes osmotic stress and second it disrupts nutrient balance (Munns and Tester, 2008). In both the cases, production of ROS is increased due to loss of coordination between different metabolic pathways (Asada, 2006). ROS causes a major disturbance in intracellular ionic homeostasis by direct activation of NSCC channels leading to additional K^+^ eﬄux besides Na-induced membrane depolarization (Demidchik et al., 2010). This activation leads to decline in cytosolic K content followed by activation of caspase-like proteases and endonucleases (Shabala, 2009; Demidchik et al., 2010) and consequently programmed cell death (Demidchik et al., 2014). ROS causes oxidative damage to proteins, lipids and DNA and thus, affect the cell membrane integrity, enzyme activities and function of photosynthetic apparatus (Yu et al., 2011). In plants, however, ROS may be considered as signaling molecules to increase antioxidant enzymes for adapting to high salt levels (Abogadallah, 2010; Jaspers and Kangasjärvi, 2010). To cope with excess accumulation of these molecules, plants have evolved a complex antioxidant defense system containing both enzymatic and non-enzymatic components. Various antioxidant enzymes that play role in maintaining the beneficial role of ROS includes SOD, dehydroascrobate reductase (DHAR), APX, Trx, Prx, GPX and GST etc. (Yu et al., 2011; Bose et al., 2013; Wang et al., 2013). The function of SOD is to catalyze the conversion of O_2_^-^ to H_2_O and O_2_ during various stresses, which is deemed to be one of the first lines of defense against free radical damage in plant cells. Accumulation of SOD in response to salt stress has been reported to play a protective role in *Canola*, *S. europaea*, and *Solanum chilense* (Wang et al., 2009; Zhou et al., 2011). In a study conducted on *K. candel* (Wang et al., 2013), SOD levels have increased in response to salinity. Similar results have been reported by Cheng et al. (2015) in *Tangut nitraria* whereas in *Puccinellia tenuiflora* (Yu et al., 2011) its activity have slightly increased under 50 mM NaCl but dramatically decreased under 150 mM NaCl treatment. DHAR protein in previous reports has showed up-regulation under salt stress. Increase in DHAR abundance may be a result of H_2_O removal through the production of ascorbic acid during stress. DHAR is frequently designated as an enzyme to protect against oxidative stress in plants. Previous studies have indicated that the antioxidative defense system as a whole have been induced during salt stress for scavenging ROS (Jithesh et al., 2006; Abogadallah, 2010). Furthermore, expression levels of antioxidant enzymes APX, Trx, Prx, GPX, and GST decreased in *Puccinellia tenuiflora* (Yu et al., 2011) but enhanced in *Tangut nitraria* (Cheng et al., 2015) under salinity condition. Yu et al. (2011) have reported in *Puccinellia tenuiflora* that the halophyte cope with ill effects of salinity by enhancing photorespiration. Also, levels of GO and CAT have increased, enhancing the oxidation of glycolate and scavenging of H_2_O_2_. Moreover, a photosynthetic enzyme, Ferredoxin—NADP(+) reductase (FNR), has been proposed to participate in a variety of redox pathways and has been confirmed to function in defense against oxidative damage (Krapp et al., 1997). The increase of FNR in *P. tenuiflora* leaves has enhanced the redox function but not photosynthesis since photosynthesis has been decreased under salt treatment (Yu et al., 2011). Similarly, FNR activity increased in *Tangut nitraria* (Cheng et al., 2015).

### Chaperones

Salt stress results in protein misfolding or unfolding, which injures plant cells. To avoid these, cells produce molecular chaperones, such as the members of HSPs, which assist protein folding or assembly and prevent irreversible protein aggregation by maintaining native conformations during salt stress (Sun et al., 2001; Pang et al., 2010). Under adverse conditions, HSPs can protect plants against stress by refolding proteins to re-establish normal protein conformation and maintain cellular homeostasis. Previous studies have reported that HSP 70 have been implicated in a variety of cellular processes, including the folding of nascent chain polypeptides or the import/translocation of mitochondrial or chloroplast precursor proteins. Role of HSP 70 in response to salt stress has been reported in *Tangut nitraria*, *Aeluropus lagopoides*, and *Physcomitrella patens* (Wang et al., 2008b; Cheng et al., 2015; Sobhanian et al., 2010). Another report have indicated that HSP 60, a mitochondrial chaperone, plays a vital role in the transport of proteins from the cytoplasm into the mitochondrial matrix and in the refolding of proteins, thus preventing protein aggregation when the mitochondria are subjected to stress (Rospert et al., 1996). Increase in expression of HSP 60 has been reported in *Physcomitrella patens* (Wang et al., 2008b).

### Proteins Involved in Signal Transduction

One of the proteins that increased abundantly during salt stress is a 14-3-3-like protein. In plants, the 14-3-3 proteins, a highly conserved family, are known to be involved in responses to diverse stresses including salinity (Zhang et al., 2013). The biological roles of 14-3-3 complexes are in the regulation of primary metabolism, signal transduction, and subcellular and cell defense reactions. They are also recognized as positive regulators of plasma membrane H-ATPase in the regulation of ion transport and cytoplasmic pH (Finnie et al., 1999). Moreover, 14-3-3 proteins have been implicated in various signal transduction pathways through controlling the activities of kinases and phosphatases, which suggests that 14-3-3 proteins regulate multiple pathways involved in salt stress responses in higher plants. In *Physcomitrella patens*, it has been reported that 14-3-3 protein have up regulated in response to salinity along with phototropin. They both work in synchronization to regulate the activity of plasma membrane H^+^ ATPase for the opening of stomata and ion channels (Wang et al., 2008b). Also, its activity has been reported in *Tangut nitraria* (Cheng et al., 2015). Another molecule, CRT has been up-regulated in response to salt treatment. CRT is one of the most important calcium-binding proteins which are involved in calcium signaling in the endoplasmic reticulum during the salt stress response in plants (Persson et al., 2003). Therefore, the results have indicated that up-regulation of CRT might play roles in signal transduction in various halophytes under salt stress (Wang et al., 2008b, 2013; Sobhanian et al., 2011; Cheng et al., 2015).

### Proteometabolism

Proteasome, which involves in regulating the particular protein concentration, can degrade unneeded or damaged proteins in plant cells. Disulfide isomerase (DI) is an enzyme that participates in disulfide bonds formation and breakage between cysteine residues when proteins folds (Wilkinson and Gilbert, 2004; Gruber et al., 2006). The up-regulation of DI has been noted in *K. candel* (Wang et al., 2013). DI causes the rearrangement of disulfide bonds in a single protein that exist intra-molecularly. In *K. candel* proteasome subunit beta type 6, 9, has been up-regulated by 300 and 450 mM NaCl but down-regulated by 600 mM NaCl. Similarly, 26s proteasome regulatory subunit 7 has up-regulated in *Tangut nitraria* at high salt concentration (Cheng et al., 2015). This suggests that degradation of unneeded, damaged, and misfolded proteins by the proteasome pathway have active role in plant resistance to salt toxicity.

## Metabolome Analysis in Halophytes

Metabolomics is one of the most rapidly advancing analytical approaches that aim at the comprehensive analysis of a large numbers of metabolites (Liu et al., 2011). It is most extensively utilized method in the field of plant sciences, since plants possess the unique feature of synthesizing a large number of natural products that do not exist in any other life form. Metabolome refers to the complete set of small-molecule metabolites (such as metabolic intermediates, hormones and other signaling molecules and secondary metabolites) to be found within a biological sample (Brosché et al., 2005). The word has been coined in analogy with transcriptomics and proteomics; like the transcriptome and the proteome, the metabolome is dynamic, changing from second to second. Metabolites, bioactive compounds, or compatible solutes are the biomolecules produced by plants naturally and also under stressed conditions. In plant-based metabolomics, metabolites can be “primary” and “secondary” metabolites. A primary metabolite directly regulates normal growth, development, and reproduction whereas a secondary metabolite is not directly involved in those processes, but usually has important ecological function.

Salt stress leads to severe osmotic disbalance, causing detrimental changes at physiological and molecular level in cellular components (Vinocur and Altman, 2005). To combat with such conditions, plants responses with up and down regulation of wide range of metabolites which protects them and prevents the detrimental effects of salinity. A wide range of metabolites has been identified, including mono-, di-, oligo-, and polysaccharides, such as glucose, fructose, sucrose, trehalose, raffinose, and fructans; amino acids, such as proline, pipecolic acid; methylated proline-related compounds, such as methyl-proline, proline betaine, and hydroxyproline betaine; sugar alcohols (polyols) such as sorbitol, mannitol, glycerol, inositol, and methylated inositols; other betaines, such as GB, b-alanine betaine, choline *O*-sulfate; and tertiary sulphonium compounds, such as dimethylsulphoniopropionate (DMSP), in halophytes in response to salinity (Rhodes and Hanson, 1993; Ashraf and Foolad, 2007; **Figure 1**, **Table 3**). In the following sections, the most common metabolites in halophytes are described in detail.

**Table 3:** Salinity induced regulation of major metabolites in different halophytes.

Metabolite	Plant species	Regulation		Function	Reference
		Up	Down		
Polyols straight chain (Mannitol and Sorbitol)	*Mesembryanthemum crystallinum* (Ice plant)	↑	–	Osmotic adjustments and osmoprotection	Loewus and Dickinson (1982)
Polyols cyclic (*myo*-inositol)	*M. crystallinum* (Ice plant)	↑	–	Osmotic adjustments and osmoprotection	Loewus and Dickinson (1982)
Phytate	*M. crystallinum* (Ice plant)	↑	–	Serves as phosphate storage foe seed	Yamaguchi-Shlnozakl and Shinozaki (1995)
Abscisic acid (ABA)	*M. crystallinum* (Ice plant)	↑	–	Regulates growth, Promotes switch from C_3_ to Crassulacean acid metabolism (CAM)	Thomas et al. (1992), Thomas and Bohnert (1993), Yamaguchi-Shlnozakl and Shinozaki (1995)
	*Arabidopsis*	↑	–	Regulates growth	Thomas and Bohnert (1993), Yamaguchi-Shlnozakl and Shinozaki (1995)
IMT1	*Porteresia coarctata*	↑	–	Pinitol synthesis	Agarie et al. (2009)
D-pinitol	*Acrostichum aureum*	↑	–	Osmoprotectant	Sun et al. (1999)
β-alanine betaine	*Populus euphratica*	↑	–	Osmoprotectant	Brosché et al. (2005)
	*Sueada salsa*	↑	–	Osmoprotectant	Liu et al. (2011)
Prolines	*Olea europaea*	↑	–	Promotes the activity of antioxidant enzymes, photosynthesis and plant growth	Ahmed et al. (2010)
	*P. euphratica*	↑	–	Osmoprotectant	Brosché et al. (2005)
	*Thellungiella halophila*	↑	–	Osmoprotecttant and ROS scavenging	Gong et al. (2005)
Sucrose, fructose, and glucose	*T. halophila*	↑	–	Osmoprotecttant and ROS scavenging	Gong et al. (2005)
Tyrosine	*S. salsa*	↑	–	Osmoprotectants and protection of PSII	Liu et al. (2011)
Organic acids	*Limonium latifolium*	–	↓		Gagneul et al. (2007)
2-Methyl-malic acid and Malonic acid	Lotus *creticus*	↑	–	Osmoprotectent	Sanchez et al. (2011)
Citric, succinic, fumaric,	*L. creticus*	–	↓	Osmoprotectent	Sanchez et al. (2011)
INO1	*P. coarctata*	↑	–	Pinitol synthesis	Agarie et al. (2009)
Conjugated polyamines	*Thellungiella salsuginea*	↑	–	ROS scavenging	Mapelli et al. (2008)

### Metabolites Related to Amino Acids Biosynthesis

Metabolome analysis showed that amino acid biosynthesis increased in response to salinity stress (Brosché et al., 2005; Gagneul et al., 2007; Sanchez et al., 2008; Liu et al., 2011). Amino acids play major role in osmoregulation during salt stress (Slama et al., 2015). In addition, they protect macromolecular sub-cellular structures and mitigate oxidative damage caused by free radicals produced in response to salt stress (Hasegawa et al., 2000). Elevated levels of amino acids and organic acids accumulation in plant cells have been correlated with enhanced stress tolerance through the scavenging of free radicals and protecting enzymes (Flowers and Colmer, 2015). Role of amino acids and organic acid in conferring salt stress is elaborated here.

#### Amino Acids and Organic Acids

It has been reported that amino acids, such as proline, tyrosine, alanine, cysteine, arginine, glycine, amides such as glutamine and asparagine, and the non-protein amino acids c-aminobutyric acid, pipecolic acid, citrulline and ornithine are accumulated in halophytes under conditions of high salinity (Mansour, 2000; Ahmad and Prasad, 2012). Amino acids such as cysteine, arginine, and methionine, which constitute about 55% of total free amino acids, have decreased when exposed to salinity stress, whereas proline accumulation is a well-known measure adopted for alleviation during and after on-set of salinity stress (El-Shintinawy and El-Shourbagy, 2001; Matysik et al., 2002; Ahmed et al., 2010; Saxena et al., 2013). Proline accumulation is a common immediate response to salt stress (Lokhande and Suprasanna, 2012).

Proline is low-molecular-weight chaperone which act as an osmoprotectants. It functions by reducing the inhibitory effects of Na^+^ ions on various enzyme activities. Thus, increases the stability of enzymes preventing the dissociation of enzyme complexes such as the OEC of PSII (Hasegawa et al., 2000). This stress responsive amino acid is predominantly synthesized from glutamate using two important enzymes such as pyrroline-5-carboxylate synthetase (P5CS) and pyrroline-5-carboxylate reductase (P5CR) (Hu et al., 1992). In osmotically stressed cell glutamate functions as the primary precursor (Gupta and Huang, 2014). Different kinds of environmental stress leads to accumulation of proline at different rates for example, in cells of *Distichlis spicata* treated with 200 mM NaCl, the cytosolic proline concentration has been estimated to be more than 230 mM (Ketchum et al., 1991). Proline has also shown to have negative impacts on some plants especially in some glycopytes for example; in *Arabidopsis* Lv et al. (2011) have reported that the proline production leads to cell toxicity under high temperature conditions. Wang et al. (2008b) previously have reported that proline accumulate to high levels in Na^+^ or osmotically stressed *P. euphratica*. Naidu (2003) has reported that some of the halophytes produced proline analog, for example *Melanleuca bracteata* accumulated the proline analog 4-hydroxy-*N*-methyl proline (MHP). Such proline analogs adds to the ability of plants to survive during salinity stress by helping in regulation, compartmentalization, and production outlay (Aslam et al., 2011). Proline is also involved in maintaining the NADP^+^/NADPH ratios levels required for normal metabolism (Hare et al., 1998). Under salinity stress proline might sever as a sink for excess reductants necessary for maintenance of photosynthetic and respiratory processes. High concentrations of NADP^+^ are necessary for regeneration of NADPH and synthesis of purines (Slama et al., 2015). Similarly, Liu et al. (2011) have reported tyrosine as an abundant amino acid in the *Suaeda salsa* compared with other amino acids such as glycine, glutamate, valine, leucine, and isoleucine. In plants, tyrosine is produced by an intermediate on the shikimate pathway called as prephenate. Prephenate is oxidatively decarboxylated with retention of the hydroxyl group to give *p*-hydroxy phenyl pyruvate, which is transaminated using glutamate as the nitrogen source to give tyrosine and a keto-glutarate. A tyrosine residue plays an important role in chloroplasts (PSII); it acts as an electron donor in the reduction of oxidized chlorophyll (Wang et al., 2013). Sanchez et al. (2011) have previously identified in Lotus, the amino acids such as proline, serine, threonine, glycine, and phenylalanine increased to protect against high salinity. Similarly in halophytic species *Limonium latifolium* organic acids have decreased upon salt stress (Gagneul et al., 2007). A comparative study by Sanchez et al. (2008) showed that citric, malic and succinic acids have expressed constitutively higher in *T. halophilla* than in *A. thaliana*. A recent comparative study on *Lotus creticus* and its related glycophytes have disclosed that 2-methyl-malic acid and malonic acid have been among the highly discriminatory metabolites, and their levels have found to be lower and higher respectively (Sanchez et al., 2011). Also, In *L. creticus* it has been noted that organic acids such as citric, succinic, fumaric, erythronic, glycolic, and aconitic acid have decreased in response to salinity (Sanchez et al., 2008).

#### Quaternary Ammonium Compound

Analogous to organic acids up and down regulation the quaternary ammonium compounds (QACs) also accumulate in plants subjected to salt stress. These include glycinebetaine, β-alanine betaine, proline betaine, choline *O*-sulfate, hydroxyproline betaine, and pipecolate betaine (Rhodes and Hanson, 1993; Mansour, 2000; Ashraf and Harris, 2004; Chen and Murata, 2008). Organic osmolytes are substances synthesized or taken from the environment by the plant in order to protect itself from stress condition. Betaine usually serves as an organic osmolyte. It has been reported that betaine is an important secondary metabolite for the protection from osmotic stress in *S. salsa* (Liu et al., 2011). The pathway of betaine synthesis is short and straight forward: choline mono oxygenase (CMO) converts choline (a detectable metabolite in *S. salsa*), to betaine aldehyde, and betaine aldehyde dehydrogenase converts this product to betaine.

Among the variety of QACs, GB is one of the most abundantly occurring in plants exposed to dehydration due to salinity (Lokhande and Suprasanna, 2012). The accumulation of GB (>90 μmol dry weight) has been well documented in many halophytic species (Rhodes and Hanson, 1993; Flowers and Colmer, 2008). Many studies have confirmed that relatively low concentrations of GB (<5 μmol dry weight) can protect the membrane and photosynthetic apparatus during abiotic stresses (Fitzgerald et al., 2009). GB is synthesized at higher concentrations in the plants when exposed to dehydration stress (Lokhande and Suprasanna, 2012). The main role of GB is to stabilize the protein quaternary structure and reduce lipid peroxidation during and after on-set of salinity stress (Mahajan and Tuteja, 2005; Chinnusamy et al., 2006; Chen and Murata, 2008). In several plant species, a positive correlation between leaf osmotic potential and GB has been observed (Rhodes and Hanson, 1993). GB is located in chloroplast where it plays an important role in osmotic adjustment and protection of thylakoid membrane, by maintaining the photosynthetic machinery in active state (Genard et al., 1991; Ashraf and Foolad, 2007). GB is also known to stabilize the association of extrinsic PSII complex protecting the PSII complex under salt stress (Murata et al., 1992). Metabolomic studies have shown that GB synthesis-associated CMOs are induced in salt treated *S. salsa* (Li et al., 2011), *S. europaea* (Wang et al., 2009) and *S. aegyptiaca* (Askari et al., 2006). Dimethylglycine (DMG) and dimethylamine (DMA) have shown relatively high abundances in *S. salsa*. DMG is an amino acid (glycine) derivative like betaine found in the cells of all plants (Li et al., 2011). Besides GB, β-alanine betaine also acts as an osmoprotectant under saline condition. It has been proposed by Hanson et al. (1991) that β-alanine betaine is a more suitable osmoprotectant than GB under saline conditions because the first step in GB synthesis requires molecular oxygen and also β-alanine betaine is synthesized from the ubiquitous primary metabolite β-alanine, reducing the metabolic competition for choline (Hanson et al., 1991; Duhaze et al., 2003). Proline betaine, a dimethyl proline also called stachydrine, also accumulates in a few halophytic species of the Plumbaginaceae, Capparidaceae, Myrtaceae, Rutaceae, Labiatae, Compositae and Leguminosae (Rhodes and Hanson, 1993; Naidu, 2003).

### Metabolites of Carbohydrate Metaboloism

#### Sugars

Accumulation of sugars has been associated with salinity and drought tolerance mechanism as accumulation of compatible solutes results in increase in cellular osmolarity leading to influx of water into the cell or at least reducing eﬄux from the cell and thus maintaining the turgor necessary for expansion of the cell (Hare et al., 1998). The major role played by these carbohydrates in stress mitigation involves osmoprotection, carbon storage, and scavenging of ROS. Sucrose, as a compound of energy storage in plant have been reported much higher than fructose and glucose and was ∼5–10% of betaine in the NMR spectral intensities from *S. salsa* samples (Li et al., 2011). According to Sanchez et al. (2008) sucrose, fructose and glucose have shown higher concentrarion in *T. halophila* in response to high salinity. Similar findings have been reported in *Juncus acutus* and *Juncus maritimus*, where sucrose, glucose, and fructose accumulated in response to salinity but sucrose have accumulated in higher amounts than glucose and fructose (Gil et al., 2011). In the same context, decrease in starch content and increase in reducing and non-reducing sugar content have been noted in leaves of *Bruguiera parviflora* (Parida and Das, 2005).

#### Sugar Alcohols

Sugar alcohols or polyols are compounds containing multiple hydroxyl functional groups available for organic reactions. They can be classified into two major types, cyclic (e.g., pinitol) and acyclic (e.g., mannitol) (Gupta and Huang, 2014). During stress conditions polyhydric alcohol/polyols such as pinitol, myo-inositol, mannitol or ononitol accumulate for survival of the plant in that condition (Williamson et al., 2002). Polyols mostly act as compatible solutes, low molecular weight chaperons and as scavengers of stress induced ROS. *In vitro* studies have suggested the ROS scavenging capacity of poyols in the order pinitol ≈ ononitol > myo-inositol > mannitol ≈ sorbitol (Orthen et al., 1994). Subsequent studies by Agarie et al. (2009) have demonstrated that pinitol extract from *M. crystallinum* showed higher scavenging of DPPH (1, 1-diphenyl-2-picrylhydrazl) than *Lactuca sativa*, a glycophyte. In addition to this, polyols also interact with proteins, enzymes, and membranes for protection of cellular structures and in signaling (Valluru and Van den Ende, 2011).

In majority of mangrove plants, the major polyol to act as compatible solute have been pinitol as its concentration on per plant water basis have ranged from 40 to 230 mM (Sengupta et al., 2008; Parida and Jha, 2010). Pinitol is synthesized from glucose-6 phosphate. The reaction is catalyzed by MIPS (l-myo-inositol 1-phosphate synthase; gene INO1or INPS1) and IMT (inositol *O*-methyl transferase; gene IMT1) (Sengupta and Majumder, 2010). Homologs of INO1 and IMT1 have been reported in *P. coarctata* and *M. crystallinum* (Agarie et al., 2009; Sengupta and Majumder, 2010). Myo-inositol, a product of enzyme MIPS, play key role in symport of Na^+^ in shoot of *M. crystallinum* (Sengupta et al., 2008) and it is the substrate for production of pinitol by IMT1. Until now IMT1 has been characterized in *M. crystallinum* and *P. coarctata* (Sengupta et al., 2008; Sengupta and Majumder, 2010). Brosché et al. (2005) have reported that galactinol corresponding biosynthesis gene galactinol synthase have shown higher transcript levels in *Populus euphratica* on exposure to high salinity. Galactinol itself did not display any significant increase in the leaves as it is a precursor of raffinose, which has shown higher level of expression (Brosché et al., 2005).

### Polyamines

Polyamines are polyvalent compounds containing two or more amino groups and known to be stimulated by various factors such as potassium deficiency, osmotic stress, low pH, nutrient deficiency or light in plants (Walden et al., 1997; Sairam and Tyagi, 2004). Polyamines are reported to be involved in growth and development, signaling, gene expression and stress tolerance (Kuznetsov et al., 2002; Takahashi and Kakehi, 2010). The most commonly occurring polyamines are putrescine, spermidine, and spermine, with the diamines, diaminopropane and cadaverine are less commonly occurring polyamine in plants under stress (Mansour, 2000). Polyamines play major role in ROS homeostasis. It affects ROS homeostasis in two ways (Takahashi and Kakehi, 2010). First, accumulation of ployamine scavenges free redicals and activates antioxidant enzymes, decreasing ROS production. However, antioxidant properties of conjugated polyamines are more than free polyamines (Kuznetsov et al., 2002). Studies on *M. crystallinum* and *Thellungiella salsuginea* have shown that conjugated polyamines have accumulated at a greater rate than in glycophytes *Plantago major* and *Geum urbanum*, upon exposure to stress (Mapelli et al., 2008). Secondly, through catabolism in the apoplast, polyamines increase the production of ROS for hydroxyl radical-induced K^+^ eﬄux (Zepeda-Jazo et al., 2011) which has been correlated with salinity stress tolerance (Velarde-Buendía et al., 2012). Thus, halophytes maintain high level of conjugated polyamines and a low level of free polyamines during salt stress. A study by Radyukina et al. (2007) on *T. salsuginea* have showed an initial increase in free ployamine followed by decrease, thus suggesting polyamine signaling may be necessary part of salt tolerance in halophytes. Some studies on the metabolite profiling of Lotus in response to salinity have showed that levels of amines such as ethanolamine and putrescine decreased (Sanchez et al., 2011). Along with this, salinity induced elevation of polyamine is essential for efficient vacuolar Na^+^ sequestration. Polyamines efficiently block SV and FV channels preventing back leakage of Na into cytosol (Pottosin and Shabala, 2014).

### Metabolites Related to ATP Biosynthesis

ATP is vital for many biosynthetic pathways in the cell. It is well known that energy requirement increases under stress. So, metabolites associated with ATP generation have been reported to be up-regulated in many halophytic species like *Aeluropus lagopoides, Tangut nitraria, Sueada salsa* (Sobhanian et al., 2010; Li et al., 2011; Cheng et al., 2015). Under salt stress enhanced expression of Adenine, adenosine, and adenosine 5′-monophosphate have been documented in *A. lagopoides* suggesting high demand of energy under stress. Also, enhancement in levels of PPP has been observed in *P. tenuiflora*. Enhanced expression of PPP gives more G3P, G6P, NADPH, and erythrose-4-phosphate (E4P) to produce larger amount of ATP. In addition, caffeic acid *O*-methyltransferase (COMT) involved in phenylpropanoid metabolism have increased in salt-treated *P. tenuiflora.* Based on these studies, changes in ATP levels is in response to declined photosynthesis and increased need for osmotic adjustment in high saline condition. Thus, it can be concluded from these studies that capacity of ATP biosynthesis is maintained in halophytes to supply energy survival and tolerating the salt concentrations.

### Metabolites of TCA Cycle, Calvin Cycle, and Glycolysis

Since, the need for amino acids increases under salinity, the metabolites of TCA cycle is used up for production of higher amount of amino acids. It has been reported in *Aeluropus lagopoides* that expression levels of CIT, aconitate, 2-oxoglutarate, SUC and fumarate decreased at high salt concentrations (Sobhanian et al., 2010). A similar trend has been observed in *S. salsa* where levels of CIT and SUC declined at high salinity (Li et al., 2011). Like TCA cycle, metabolites of Calvin cycle like Ribulose 5-phosphate, Ribose 5-phosphate and Glucose 6-phosphate, have also declined to compensate for the decrease in photosynthetic rate and increased energy demand (Sobhanian et al., 2011; Wang et al., 2013). Pyruvate, a metabolite of Glycolysis pathway, also showed decreased expression as increased in demand of amino acids like proline which have used up the pyruvate in TCA cycle. This has been reported in *Aeluropus lagopoides* by Sobhanian et al. (2010).

### Metabolites Associated with Detoxification and Antioxidation

A greater ability of halophytes to tolerate high levels of salinity has been attributed to their higher antioxidant capacity compared to glycophytes (Flowers and Colmer, 2008; Kosová et al., 2013; Ozgur et al., 2013). Antioxidant metabolism, which includes antioxidant enzymes and non-enzymatic compounds, play a critical role in detoxifying ROS induced by salinity stress. Salinity tolerance is positively correlated with the activity of antioxidant enzymes, such as SOD, CAT, APX, GPX, and GR (Gupta and Huang, 2014). Along with these accumulations of non-enzymatic antioxidant compounds have also shown antioxidative ability (Bose et al., 2013). Many compatible solutes like proline, Polyamines, GB, polyols etc, have been considered as non-enzymatic antioxidants due to their ability to scavenge HO^⋅^. Proline, has been shown to perform many antioxidant functions such as quenching of H_2_O_2_ and HO^⋅^, stabilization of ROS scavenging enzymes, protection of complex II of mitochondrial electron transport chain and preventing PCD during stress signaling, adaptation, and recovery (Bose et al., 2013). Cuin and Shabala (2007) have shown that exogenous application of proline decreases hydroxyl radical induced K^+^ eﬄux from *Arabidopsis* roots (Cuin and Shabala, 2007). As discussed earlier in the review polyols such as sorbitol, mannitol, myo-inositol, pinitol accumulate in large quantities during environmental stresses (Williamson et al., 2002). Apart from being used for osmotic adjustment, these molecules are also efficient hydroxyl radical scavengers. Subsequent experiments showed that a pinitol extract from the halophyte *M. crystallinum* showed 2-fold higher scavenging of DPPH (1, 1-diphenyl-2-picrylhydrazl) than glycophyte *Lactuca sativa* (Agarie et al., 2009). In addition to direct scavenging, polyols can interact with membranes, protiens and different enzymes for stress related signaling and protection of cellular structures (Valluru and Van den Ende, 2011).

### Role of Phytohormones in Salt Tolerance

Phytohormones are chemical messengers, which play critical roles in regulating plant responses to stress at extremely low concentration. Changes in level of phytohormones like jasmonic acid (JA), gibberellin (GA), ethylene (ET) and ABA and enzymes related to their biosynthesis like allene oxide cyclase (AOC); lipoxygenase (LOX) (JA biosynthesis), DWARF3 (GA biosynthesis), SAMS (ET biosynthesis), NCED (ABA biosynthesis) have been reported in many halophytic species in response to high salt concentration. The main phytohormone which shows abundance in expression is ABA. ABA is a major internal signal induced during adverse environmental conditions such as salt stress for survival of the plant. An increase in ABA concentration has been noticed in plants on exposure to salinity. In most cases, it is correlated with water potential of leaf or soil suggesting that salinity-induced increase in endogenous ABA is due to water deficit rather than specific salt effect (Zhang et al., 2006). Salt stress induces abundance of ABA biosynthesis as increase in ABA biosynthesis enzyme NCED level has been documented in *Thellungiella salsuginea* (Taji et al., 2004). The halophyte has showed an increase in expression of many early and delayed ABA responsive genes (ABA-dependent transcription factors, e.g., Lea/Rab genes). ABA plays dual role in response to salt stress. First, an immediate ABA response directs a decrease in pH causing loss of turgor in stomatal guard cells leading to stomatal closure. Second, a delayed ABA response includes induction of many ABA responsive transcription factors called as early response genes. These factors bind to ABA responsive promoter elements (ABRE) in the promoters of delayed response genes like Lea/Rab genes. The products of these genes accumulate in plant cells to high levels to confer high salt tolerance (Taji et al., 2004). ABA receptor includes PYR/PYL/RCAR proteins. These proteins contain steroidogenic acute regulatory StAR—related lipid transfer (START) domain which binds to small hydrophobic molecules like Ca^2+^, using it as a secondary messenger. Like ABA, JA biosynthesis have also enhanced in *A. thaliana* showing increase in JA induced signaling at high salt concentration (Jiang et al., 2007; Pang et al., 2010). GA is another important hormone which activates many vital processes to cope with stress. DWARF3 gene expression has been noted high in *Triticum aestivum/Thinopyrum ponticum* hybrid under salt conditions. This gene is involved in GA biosynthesis. Thus, high expression of GA is important for revealing stress and senescence (Jiang et al., 2007). In general, JA and ET signaling pathways have been found to be involved in responses to salinity stress. Earlier studies have revealed increase in expression of salt-regulated mitogen-activated protein kinases (MAPKs) and salt responsive jasmonate-inducible proteins in *T. halophila, P. patens*, and *S. europaea* (Wang et al., 2008b; Pang et al., 2010).

## Ionomics Studies in Halophytes

Ionomics is the study of ionome, where ionome is defined as the total inorganic component of cellular and living systems (Satismruti et al., 2013). It comprises of mineral nutrient and trace element composition of an organism. Ionomics includes the quantitative measurement of the elemental composition of an organism (Salt et al., 2008). It also considers the changes in mineral composition in response to physiological stimuli, developmental state, genetic modifications and stress tolerance. Ionomics utilizes high-throughput elemental analysis technologies integrated with genetic and bioinformatics tools. It provides information about the functional state of an organism in response to different biotic and abiotic factors. Ionomic analysis provides a powerful approach for the functional analysis of the genes and gene networks that control the ionome and physiological processes that are indirectly involved in controlling ionome (Baxter, 2010). Ionomics gives high throughput results involving low cost, thus provides an easy means of analysis. Different analytical tools used for total ionomic profiling of plants are ICP-MS, ICP-OES, X-Ray crystallography, Neutron Activation Analysis (NAA) etc. Purdue Ionomics Information Management System (PiiMS) is the database for storing all the ionic profiles of plants (Baxter et al., 2007). The data present in this database helps in various structural and functional genetic studies on an organism.

Acclimation of plants to saline soils involves changes in the uptake, transport and/or partitioning of mineral ions to reduce the toxicity caused by high salt concentration (Sanchez et al., 2008). For normal cellular function in plants a high K^+^/Na^+^ ratio is required. At higher Na^+^ concentration deficiency of K^+^ in the cytosol is a result of poor retention of K^+^ caused due to Na- induced membrane depolarization causing K^+^ eﬄux through depolarization-activated KOR channels (Shabala and Mackay, 2011; Bose et al., 2014b). In addition, K^+^ may also leak through NSCCs as a result of the accumulation of ROS during salt stress (Bose et al., 2014a). Also, Na^+^ may block K^+^ specific transporters imposing toxic effect and induces lethal changes in protein conformation (Zhu, 2003; Mahajan and Tuteja, 2005). In the saline environment, Na^+^ enters into the cell cytosol through selective and non-selective transporters and through cation channels (Sanchez et al., 2011). Halophytes adapt various mechanisms to prevent accumulation of Na^+^ in the cytoplasm which include Na^+^ extrusion and/or intracellular compartmentalization of Na^+^ along with recirculation of Na^+^ out of the shoot and up regulation of Na^+^/H^+^ antiporters in plasma membrane, (Hamada et al., 2001; Shi et al., 2003). Two halophytes, *Atriplex lentiformis* and *Chenopodium quinoa*, have been investigated for responses of root cell membrane potential and net H^+^, Na^+^ and K^+^ fluxes to NaCl-salinity showing that small depolarization of root cell plasma membrane causes fast response of plasma membrane H^+^ ATPase for restoration of plasma membrane potential and H^+^ electrochemical gradient to drive transport of Na^+^ out of the cell (Bose et al., 2014b).

Various antiporters and transporters play crucial role in balancing the Na^+^ content and removing the excess amount. Amongst these are Na^+^/H^+^ antiporters (SOS pathway) that play important role in eliminating toxic cytosolic Na^+^ from cells (Kant et al., 2006). In addition to this, compartmentation of Na^+^ in the vacuoles by K^+^/Na^+^ specific NHX1-type antiporters and maintenance of low Na^+^ in cytosol by HKT1-type transporters prevent or reduces movement of Na^+^ to aerial parts of plant. For normal cellular function optimum cytosolic K^+^/Na^+^ ratio is necessary (Zhu, 2003). Under high salt conditions, to maintain the cellular potassium level, activity or expression of potassium-specific transporters is enhanced as it has been shown in *Mesembryanthemum crystallinum L*, the up-regulation of high affinity K^+^ transporter- K^+^ uptake genes by Su et al. (2003).

With increasing accumulation of Na^+^ reduction in Ca^+^ and Mg^+^ uptake and sometimes carbon assimilation have been reported in halophytes (Parida et al., 2004). Calcium plays a significant role in signaling during salt stress. Onset of salinity causes transient increase in extracellular calcium (Zehra et al., 2012). The possibility is that extracellular Ca^+^ first alters Na^+^ influx directly and then maintains Na^+^ and K^+^ homeostasis via SOS pathway (Mahajan et al., 2008). In this context, Venkatesalu et al. (1994) have reported in *Sesuvium portulacastrum*, a salt marsh halophyte, that potassium (K), and calcium (Ca) accumulation level have increased exponentially with NaCl treatment. They have also noted that, up to 600 mM NaCl concentration, some essential elements like copper (Cu), iron (Fe), manganese (Mn) and zinc (Zn) significantly accumulated in all plant parts. Similarly, in *L. creticus*, calcium and boron accumulation have been reported low as compared to the glycophytes of the same genus but Mg, P, Fe, and Zn have accumulated in high amount (Sanchez et al., 2011). Halophyte *Aster tripolium* remarkably accumulate K^+^ for ion homoeostasis even under high NaCl stress. Also, the amounts of Ca, Fe, and Mg, but not those of P, S, Cl, and K, have been found highest in the outer root area (Scheloske et al., 2004). For survival of the plant under high salt condition maintaining the proper ion balance is necessary. Ion homeostasis is maintained by membrane transporters (Schroeder et al., 2013) like SOS1 (salt overly sensitive), NHX1 (Na^+^/H^+^ exchanger), H^+^-ATPase (Shi et al., 2002), high-affinity K^+^ transporter (Maathuis, 2006) and high-affinity K^+^ transporter (Sanadhya et al., 2015). Role of these transporters in maintaining ion homeostasis is discussed below:

### SOS1

Molecular genetic studies in various halophytes have led to the identification of a plasma membrane Na^+^/H^+^ antiporter, SOS1 which plays a vital role in removal of Na^+^ from shoot in *T. halophila* under saline conditions (Kant et al., 2006). Sodium eﬄux through SOS1 under salinity is regulated by SOS3–SOS2 kinase complex (Chinnusamy et al., 2005). Similar findings have been shown by Katschnig et al. (2015) in salt-accumulating halophyte *Salicornia dolichostachya*, where SOS1 expressed at a high level, but expression of HKT1 have not been detectable. SOS1 pathway for eﬄux of Na^+^ from the cell includes SOS1, SOS2, SOS3 and a Ca^2+^ sensor (Khan, 2011). In SOS pathway, a proton gradient is generated by the H^+^-ATPase in the plasma membrane and H^+^-PPiase in the vacuolar membrane. SOS3 perceives the extracellular salt stress signals through the Ca^2+^ signals. The SOS3, in turn activates SOS2, a serine/threonine protein kinase. The activated SOS2 then phosphorylates the SOS1, a plasma membrane Na^+^/H^+^ antiporter for transporting Na^+^ out of the cytosol. SOS2 plays important role as it also phosphorylates the vacuolar Na^+^/H^+^ antiporter for accumulation of Na^+^ inside the vacuole (Chinnusamy et al., 2005).

### HKT-Type Na^+^ Transporters

High affinity potassium transporters are carrier type proteins that mediate Na^+^ and K^+^ transport. Members of HKT family are Na^+^ specific transporters that mediate Na^+^ transport or Na^+^- K^+^ symport. HKT-type Na^+^ transporters play a significant role in keeping low cytosolic Na^+^ concentrations. HKT1 transporters functions as symporter for both Na^+^ and K^+^ and also as a selective Na^+^ uniporter. Importance of HKT1 has been shown in halophyte *M. crystallinum* and *Suaeda salsa* taking part in the maintenance of cytosolic cation homeostasis, particularly, in the maintenance of K^+^ nutrition under salinity (Su et al., 2003; Shao et al., 2014). HKT1 acts importantly in cation uptake from soil and in loading of Na^+^ to vascular tissue which in turn leads to Na^+^ accumulation in leaves of *M. crystallinum* in high salt concentration (Su et al., 2003).

### V-type H^+^- ATPase

Compartmentation of Na^+^ in the vacuole is of great importance for halophytes as it limit excessive NaCl accumulation in the symplast, thus protect enzymes, which are sensitive to high salt, in the cytoplasm and chloroplasts (Li et al., 2011). Na^+^ sequestration into the vacuole is regulated by activity of V-type H^+^- ATPase and H^+^- PPase. These phosphatases are necessary for activity of Na^+^/H^+^ antiporters as they generate the proton gradient required for influx of Na^+^ into the vacuole. V-ATPase is a type of H^+^-ATPase located on tonoplast. It is a multi heteromeric complex of at least 11 different subunits, arranged in a head/stalk/base arrangement (Shabala et al., 2014). For the formation of a proton gradient vacuolar H-ATPase transports protons across the tonoplast. Thus, providing the driving force for active Na^+^ transport into the vacuole, and reducing the toxic levels of Na in the cytoplasm. This accumulation of Na^+^ into the vacuole is an effective strategy for balancing Na^+^ concentration in cytoplasm and maintains cell osmosis in plant (Du et al., 2010). But for conferring salinity tolerance toxic Na must be prevented from leaking back into the cytosol. Here, for prevention of leaking back of Na into the cytosol slow activating (SV) and fast activating (FV) tonoplast channels play important role as reported in *Quinoa*, a facultative halophyte species (Bonales-Alatorre et al., 2013). Both the Na^+^ concentration and the expression of vacuolar H-ATPase have increased and K^+^/Na^+^ ratios only decreased slightly with increasing salt concentration as reported by Wang et al. (2009) in *Salicornia europaea* and by Wang et al. (2014) in *K. candel*. Thus, up regulation of vacuolar H-ATPase suggests that it might play a vital role in salinity tolerance. Similarly, in *M. crystallinum* an increase in activity of V-ATPase under high salt concentration have been detected by Ratajczak et al. (1994).

For elimination of excess salt from metabolically active tissue, many halophytes have developed specialized epidermal cells called as salt glands or bladders (Agarie et al., 2007; Flowers and Colmer, 2008; Shabala et al., 2014). Within halophytes, salt bladders are most common within Chenopods. Both *Atriplex* and *Quinoa* species have bladder cells on their leaf and stem surfaces (Shabala et al., 2014). Very high Na^+^ and Cl^-^ concentrations are reported for epidermal bladder cells (EBCs) in salt-stressed plants ranging between 500 and 1000 mM (Barkla et al., 2002). This high concentration of Na^+^ and Cl^-^ accumulated in EBC vacuoles is found to be osmotically adjusted by cytosolic potassium and accumulating organic osmolytes such as ononitol, pinitol, and proline (Agarie et al., 2007). It has been assumed that high tonoplast Na^+^/H^+^ antiport and V-type ATPase activity is responsible for an increase in the accumulation of sodium ions in the EBCs (Barkla et al., 2002). Role of other transporters are yet to be elucidated for potential uptake of sodium by EBCs rendering salt tolerance to halophytes.

## Conclusion

Salinity profoundly affects various aspects of plant cell structure and metabolism. Na^+^ ions in the soil are toxic to plants as they have adverse effect on enzymes in cytosol, photosynthesis, K^+^ nutrition and overall metabolism. Study of responses to salinity at proteome, metabolome, and ionic level would endorse for better understanding of physiological mechanisms underlying salt tolerance of halophytes. This review provides information on proteomic, metabolomic, and ionomic bases of salt tolerance in halophytes. Efforts have been made to provide the regulation of various proteins and metabolites to reduce the toxic effects of Na^+^ accumulation in halophytes. This review provides the recent knowledge that offers some ways for increasing salt tolerance. As it has been discussed earlier, proteins play a crucial role in making the halophytes tolerant to salt stress and minimizing the adverse effects of Na^+^. Recently, proteomics studies on halophyte tolerance to salinity have been engrossed mainly on studies of quantitative changes in total plant proteome or specific subcellular proteome like nuclear, mitochondrial, plastid proteomes. These studies have been based on comparative proteomics approach using 2-DE followed by MS analysis. Moreover, with the new advancements in proteomic approaches, it seems that study of post translational modification (PTMs), especially phosphoproteomics and redox proteomics, along with protein interactions (interactomics) will contribute to a detailed protein functional characterization which will surely help us to better understand the processes of salt tolerance procurement in halophytes. In the same manner, under salt stress halophytes have the ability to redirect ions and nutrients to domineering processes and induce adequate metabolic adjustments for survival during high salt concentration. However, this is only a simpler way of expressing but in order to achieve a higher degree of understanding; we must consider regulation of metabolites and ions as an intricate network of interconnected reactions as metabolic and ionic responses are complex and dynamic and involve the modification of more than one metabolite. Thus, studies of salt tolerance in halophytes at the physiological and molecular levels will provide us valuable information about their salt tolerance. These studies have revealed that under salt stress halophytes hire systematic mechanisms to develop salt tolerance and identification and characterization of the phenomenon of salt tolerance will direct us for improving salinity tolerance of crop plants.

Therefore, boost in omics approaches will elevate our understanding of the underlying mechanism of salt tolerance of halophytes, which will in turn help us in identifying the responsive proteins, metabolites and ions for enduring the tolerance. Hence, this information can be of great importance for improving the crop plants through genetic engineering. At the end we can conclude from this review that key proteins and metabolites that have been used for genetically engineering crop plants include downregulation of photosynthesis related proteins and metabolites, up regulation of energy metabolism and ion channels to compensate for toxic effect of Na^+^ and its eﬄux or compartmentalization. Thus, the use of the information delivered here in this review would provide basis for genetic engineering of crop plants and industrially important plants for salt tolerance and would be vital for solving world’s problem concerning food and energy.

## Conflict of Interest Statement

The authors declare that the research was conducted in the absence of any commercial or financial relationships that could be construed as a potential conflict of interest.

## Abbreviations

1,3-PGA: 1,3 bis phosphogylcerate
2PG: 2 Phosphogylcerate
3PG: 3 Phosphoglycerate
3-PGA: 3-phosphoglycerate
AH: aconitate hydratase
AKG: alpha-ketoglutarate
APX: ascorbate peroxidase
CAB: chlorophyll a/b binding protein
CAT: Catalase
CIT: Citrate
CRT: Calreticulin
D1: D1 protein
DHA: dehydroascrobate
DHAP: dihydroxy acetone phosphate
DHAR: DHA reductase
F 1,6-BP: Fructose 1,6 bis phosphate
F6P: fructose-6-phosphate
FBP: Fructose 1,6-bisphosphate
FNR: Ferredoxin—NADP(+) reductase
FRK: Fructokinase
G3P: glyceraldehydes-3-phosphate
G6P: glucose-6-phosphate
GB: Glycine betaine
GLR: glutaredoxin
GO: glycolate oxidase
GPX: glutathione peroxidase
GR: glutathione reductase
GSH: reduced glutathione
GSSG: oxidized glutathione
GST: glutathione S-transferase
HKT: High affinity potassium transporters
ICT: Isocitrate
LHC: Light harvesting complex
MAL: Malate
MD: Malate dehydrogenase
MDHA: monodehydroascorbate
MDHAR: MDHA reductase
Mg: magnesium
NAD+/NADH: nicotinamide adenine dinucleotide
NADP+/NADPH: nicotinamide adenine dinucleotide phosphate
OAA: oxaloacetic acid
OEE: oxygen-evolving enhancer protein
O-Fd: oxidised Ferredoxin
PC: plastocyanin
PEP: Phosphoenolpyruvate
PG: phosphoglycolate
PGAM: Phosphoglycerate mutase
PGK: Phosphoglycerate kinase
PPDK: pyruvate orthophosphate dikinase
PRK: phosphoribulokinase
Prx: peroxiredoxin
PSI: photosystem I
PSII: photosystem II
RCA: Rubisco activase
R-Fd: reduced Ferredoxin
Ru5P: ribulose-5-phosphate
RuBisCO: ribulose-1,5-bisphosphate carboxylase/oxygenase
RuMP: Ribulose monophosphate
RuBP: Ribulose-1,5-bisphosphate
PRPP: 5-phospho-D-ribose-1-pyrophosphate
SD: Succinate dehydrogenase
SOD: superoxidedismutase
SUC: Succinate
SucCoA: Succinyl CoA
TCA cycle: tricarboxylic acid cycle
TK: transketolase
TPI: Triose-phosphate isomerase
Trx: thioredoxin
V ATPase: Vacuolar ATPase.
